# A sandwich SERS immunoassay platform based on a single-layer Au–Ag nanobox array substrate for simultaneous detection of SCCA and survivin in serum of patients with cervical lesions†

**DOI:** 10.1039/d1ra03082e

**Published:** 2021-11-16

**Authors:** Yifan Liu, Menglin Ran, Yue Sun, Yongxin Fan, Jinghan Wang, Xiaowei Cao, Dan Lu

**Affiliations:** Institute of Translational Medicine, Medical College, Yangzhou University Yangzhou P. R. China cxw19861121@163.com ludan1968@126.com; Department of Obstetrics and Gynecology, Northern Jiangsu People's Hospital Affiliated to Yangzhou University, Yangzhou University Yangzhou P. R. China; The Yangzhou School of Clinial Medicine of Dalian Medical University Yangzhou P. R. China; Jiangsu Key Laboratory of Integrated Traditional Chinese and Western Medicine for Prevention and Treatment of Senile Diseases, Yangzhou University Yangzhou P. R. China; Jiangsu Key Laboratory of Experimental & Translational Noncoding RNA Research, Medical College, Yangzhou University Yangzhou 225001 China

## Abstract

The evaluation of tumor biomarkers in blood specimens is vital for patients with cervical lesions. Herein, an ultrasensitive surface enhanced Raman scattering (SERS) platform was proposed for simultaneous detection of cervical-lesion-related serum biomarkers. Raman reporter labeled Au–Ag nanoshells (Au–AgNSs) acted as SERS tags and an Au–Ag nanobox (Au–AgNB) array substrate prepared by the oil–water interface self-assembly method was used as a capture substrate. This single-layer Au–AgNB array substrate was proved to have exceptional uniformity by atomic force microscopy and SERS mapping. Numerous “hot spots” and specific adsorption surfaces offered by the Au–AgNB array substrate were confirmed by the finite difference time domain method, which could generate a SERS signal in electromagnetic enhancement. Binding of antigens between antibodies on Au–AgNSs and the Au–AgNB array substrate led to the formation of a sandwich-structure by the two metal nanostructures. Consequently, an ultralow detection limit of 6 pg mL^−1^ for squamous cell carcinoma antigen (SCCA) and 5 pg mL^−1^ for survivin in a wide linear logarithmic range of 10 pg mL^−1^ to 10 μg mL^−1^ was acquired. High selectivity and reproducibility with relative standard deviations of 7.701% and 6.943% were detected. Furthermore, the simultaneous detection of the two biomarkers in practical specimens was conducted, and the results were consistent with those of the enzyme-linked immunosorbent assay. This platform exhibited good robustness in the rapid and sensitive detection of SCCA and survivin, which could be a promising tool in early clinical diagnosis for different grades of cervical lesions.

## Abstract 2

A novel SERS immunoassay platform for simultaneous detection of SCCA and survivin based on Au–Ag nanoshells (Au–AgNSs) combined with a single-layer Au–AgNB array substrate was proposed in the current study.

## Graphical abstract

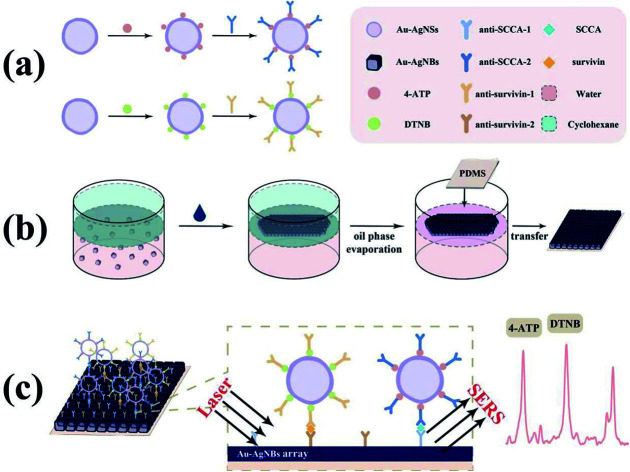

## Introduction

Cervical cancer remains one of the leading causes of death worldwide in gynecological cancer. It is estimated that 570 000 women around the world are diagnosed with cervical cancer every year.^1^ Nonetheless, cervical cancer is still the major cause of cancer-related death among the female population in 42 low-resource countries.^2^ Although significant progress has been made in cervical cancer chemotherapy, radiotherapy and surgical therapies, the mortality rate for cervical cancer is still high. On the basis of previous studies, the implementation of preventive measures can prevent the occurrence and death by cervical cancer to a large extent, and early screening is an essential part.^3,4^ In addition, only about 20% of women in low-resource have ever received cervical cancer screening compared with more than 60% in high-income countries, which is closely related to the high cost of screening.^5,6^ For half a century, the treatment of precancerous lesions found by cervical scraping cell microscopy has been the paradigm of secondary prevention of cervical cancer.^7^ Although cytology has absolutely led to a significant decrease in the burden of cervical cancer in some resource-rich countries, this method may have reached its limits.^8^ In this context, the improvement of cervical cancer screening is worth investigating. The antibodies can detect disease-related markers, and this function is widely used in early diagnosis.^9^ Squamous cell carcinoma antigen (SCCA) is a subtype of tumor-associated antigen TA-4, which exists in the cytoplasm of squamous cell carcinoma of uterus, cervix, lung, head and neck. SCCA is the first biomarker for the diagnosis of squamous cell carcinoma.^10^ Survivin is a member of apoptosis suppressor gene family, which can inhibit cell apoptosis, regulate cell cycle and promote cell proliferation.^11,12^ According to the research, the expression of SCCA and survivin in cervical squamous cell carcinoma is significantly increased, but they are rarely expressed in normal cervix and chronic cervicitis.^13,14^ In the past few decades, many analytical methods have been used to detect protein biomarkers, such as enzyme-linked immunosorbent assay (ELISA), fluorescence-based assays and electrochemical immunoassay.^15–17^ However, these techniques have the disadvantages of low sensitivity and complex procedures, which may be an obstacle in analyzing complicated specimens and real-time detection. Hence, it is imperative to develop convenient and highly sensitive methods for trace detection of SCCA and survivin in practical specimens.

Fortunately, the emergence of nanomaterials-based surface enhanced Raman scattering (SERS) technique provides a new avenue to the study of early clinical diagnosis, which is unique in high detection sensitivity, non-destructive data acquisition and intrinsic spectroscopic fingerprints.^18^ An excellent enhancement effect can be provided by roughened metal surfaces, nanoparticles with specific shapes and dimensions or regular nanostructures formed on flat substrates.^19^ Grubisha *et al.* showed that PSA for early cancer diagnosis could be detected in serum samples at very low concentrations by a SERS-based readout method.^20^ Kunushpayeva *et al.* proposed a simultaneous sandwich SERS immunoassay using Si substrates.^21^ Matteini *et al.* used concave gold nanocube assemblies as nanotraps for protein detection based on SERS.^22^ Nevertheless, SERS still has some deficiencies in clinical applications, such as the poor SERS effect of metal nanomaterials, the high price of metal SERS substrate and the complex modification process for non-metal substrates (silicon, glass, *etc.*). It can not guarantee the uniform distribution of amino or carboxyl groups on the surface after modification. Up to present, it is still a challenge to achieve uniformly enhanced SERS substrates while maintaining high sensitivity.

The intensity of SERS signal is directly related to the shape and size of nanoparticles, so the preparation of nanoparticles is an important cornerstone. The shape and high curvature characteristics of anisotropic nanoparticles can achieve multiple surface plasmon resonance peaks and local electric field enhancement (“hot spots”),^23^ which results in a SERS enhancement factor that is more pronounced than that of spherical nanoparticles.^24–26^ Among common shapes, hollow NPs, such as nanoshells, have attracted particular attention due to the higher surface area than filled nanomaterials.^27,28^ Au–Ag nanoboxes (Au–AgNBs), as a new kind of nanomaterial with unique hollow, porous wall structure and good size adjustability, have broad application prospects in diverse biomedical applications.^29,30^ The properties of SERS substrates are crucial for these applications, which amplify Raman signals by the exciting local surface plasmon resonance. However, most of capture substrates used for SERS immunoassay are mainly fabricated on two-dimensional substrates, such as glass slides or silicon wafers. Because of their flatness, they are limited in immobilizing a large number of nanoparticles. Polydimethylsiloxane (PDMS), a common polysiloxane, is an inert, avirulent, non-flammable and transparent elastic material.^31^ The efficiency of SERS on PDMS substrate rests with the size and shape of nanoparticles attached to it. Combined with ideal nanomaterials, it can induce the electromagnetic field enhancement of strong plasma nanostructures, which becomes a perfect platform for efficient SERS detection and can reach the level of single-molecule detection. It is an effective way to improve the uniformity of SERS substrate by arranging disordered nanoparticles into a single-layer of ordered-close packed films.^32,33^ Singh *et al.* deposited uniform and wide-area tilted silver nanopillar films on PDMS by the technology of oblique angle deposition (OAD).^34^ Aksu *et al.* reported nanosphere lithography (NSL) on flexible and foldable substrates.^35^ However, these technologies usually require complex operation processes, so self-assembly technology has attracted much attention because of its simplicity. The self-assembly of liquid–liquid interface is typically the formation of two-phase interface between organic phase and water phase. By controlling the surface properties of nanostructure units, they can gather at the two-phase interface to obtain the single-layer array structure. After the formation of the ordered single-layer array, the distance between the nanostructure units is reduced to less than 10 nm, and strong coupling between local fields will occur, resulting in a significant enhancement of the electromagnetic field.^36^ Li *et al.* proposed a liquid/liquid interface self-assembled monolayer film of concave star-shaped Au nanocrystals which revealed highly uniformity and repeatability.^37^ Teng *et al.* established a SERS method based on self-assembled Au nanoarrays at a liquid–liquid interface between *n*-hexane and Au colloids. Compact gold nanoparticles were conducive to forceful plasmonic coupling and the formation of electromagnetic “hot spots”.^38^ Wu *et al.* construct a novel liquid phase SERS biosensor by stabilizing Au NPs on water–oil interfaces. The functionalized self-assembled Au NP interfaces exhibited outstanding stability, tunability, and editable properties compared to randomly assembled Au NPs.^39^ Oil–water interface is becoming a powerful platform due to its bottom-up self-assembly into ordered two-dimensional and three-dimensional nanoarrays.^40^

**Fig. 1:**
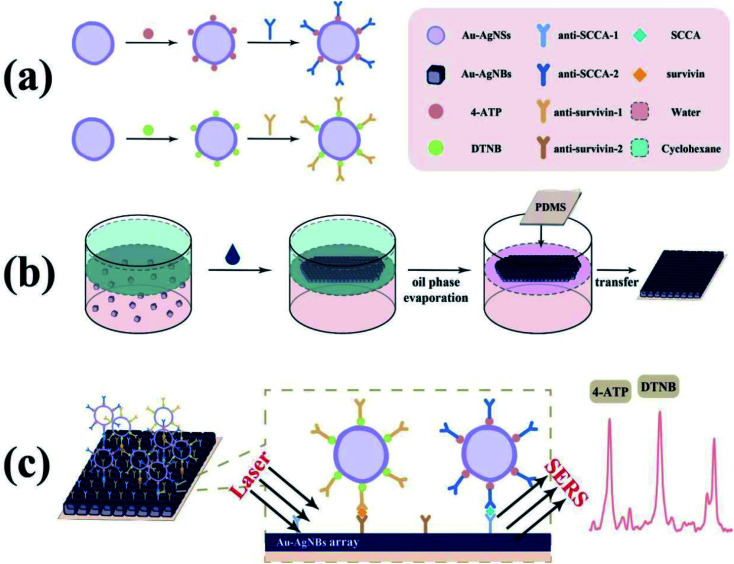
Schematic diagram (a) The synthesis procedure of 4-ATP/DTNB labeled Au–AgNSs SERS tags. (b) The fabrication of oil–water interface self-assembled Au–AgNBs array substrate. (c) Simultaneous detection of SCCA and survivin by SERS immunoassay platform.

In practical applications, the information provided by a single biomarker is inadequate for early clinical diagnosis. Hence, the simultaneous detection of multiple biomarkers has more excellent clinical value. Inspired by this, a novel SERS immunoassay platform for simultaneous detection of SCCA and survivin based on Au–Ag nanoshells (Au–AgNSs) combined with single-layer Au–AgNBs array substrate was proposed in the current study. The schematic diagram of SERS immunoassay platform for the two biomarkers simultaneous detection was revealed in Fig. 1. 5,5′-Dithiobis-(2-nitrobenzoic acid) (DTNB) and 4-aminothiophenol (4-ATP) were eminent Raman reporters that had been extensively studied and used to determine the SERS ability and conjugated for attaching antibodies to nanoparticles. Secondly, the Au–AgNBs array substrate was prepared by oil–water interface self-assembly method. The finite difference time domain (FDTD) method was used to simulate the enhancement mechanism of the Au–AgNBs array substrate. Thereafter, uniformity, sensitivity and stability of developed substrate were investigated through atomic force microscope (AFM) measurements, SERS mapping and SERS measurements. In the presence of SCCA and survivin, SERS tags were combined with capture substrate due to specific binding affinity between antigen and antibody. The signal intensities varied with different concentrations of antibody, thus realizing the detection of these two biomarkers. Subsequently, the selectivity and reproducibility of the proposed platform were assessed. Ultimately, the SCCA and survivin in clinical specimens gathered from chronic cervicitis, low-grade squamous intraepithelial lesion cervical (LSIL), high-grade squamous intraepithelial lesion (HSIL) and cervical cancer patients were quantitatively detected by the developed immunoassay platform and then compared with the results of ELISA. It is envisioned that such an attractive SERS immunoassay had brilliant potentialities for early clinical diagnosis of cervical lesions.

## Materials and methods

### Materials

Ascorbic acid (AA), silver nitrate (AgNO_3_), chloroauric acid tetrahydrate (HAuCl_4_·4H_2_O), ethanol, *n*-hexane, acetone, potassium carbonate, tetrakishydroxy-methylphosphonium (THPC, 80% aqueous solution) and poly(*n*-vinylpyrrolidone) (PVP) were all purchased from Feichang Chemicals Co. Ltd. (Yangzhou, China). Phosphate-buffered saline (PBS), *N*-hydroxysuccinimide (NHS), 4-aminothiophenol (4-ATP) and dimercaptosuccinic acid (DMSA) were all purchased from Noah Chemical Inc. (Yangzhou, China). 1-Ethyl-3-(3-dimethylaminopropyl)carbodiimide (EDC), 5,5′-dithiobis-(2-nitrobenzoic acid) (DTNB) and bovine serum albumin (BSA) were all purchased from Younuo Chemicals Inc. (Yangzhou, China). SCCA, survivin, SCCA monoclonal antibody (labeling), survivin monoclonal antibody (labeling), SCCA polyclonal antibody (coating), survivin polyclonal antibody (coating) and ELISA kits were all purchased from Sangon Biotech (Shanghai, China). Deionized water (resistivity > 18.2 MΩ cm) was used for the preparation of the specimens and throughout all the experiments. All glassware was dipped in aqua regia [HNO_3_ : HCl = 1 : 3 (v/v)] for over 24 h and washed with deionized water.

### The collection and storage of serum specimens

The serum specimens used in the experiment were provided by the College of Clinical Medicine of Yangzhou University and the specimen donors had signed the informed consent. The serum specimens were divided into four groups composed of 40 chronic cervicitis patients, 40 LSIL patients, 40 HSIL patients and 40 cervical cancer patients. Table 1 summarizes the specific information of all participants who enrolled in this study. After centrifugation (2500 rpm, 15 min, 5 °C), the serum specimens were collected and cryopreserved at −80 °C before analysis.

**Table 1:** Basic characteristics of the included subjects

Groups	Chronic cervicitis	LSIL	HSIL	Cervical cancer
Age (mean)	41	42	48	54
Specimen	40	40	40	40

### Synthesis of Au–AgNSs

The Au–AgNSs were synthesized *via* a seed-mediated growth route.^41^ In a typical synthesis, 0.025 g of potassium carbonate and 2 mL of 1% AgNO_3_ solution were placed in a flask to obtain 0.5 mM silver stock solution. At which point, 2 mL prepared THPC was quickly added to silver seeds with vigorous stirring. After stirring thoroughly for at least 10 min, 0.5 mL AA (100 mM) was injected rapidly into the above solution and a color change from yellow to blue occurred. Afterwards, Au–AgNSs were centrifuged (5000 rpm, 15 min) and re-dispersed into deionized water to remove unreacted chemicals. Secondly, 0.5 mM solution of HAuCl_4_ was prepared by dissolving 0.01 g of HAuCl_4_ in a mixture of 100 mL of water and 5 mL 1% PVP solution. 5 mL of the above solution was quickly dropped into Ag nanoshells solution with mild stirring and the mixture continued to react for 2 h. After cooling it to room temperature, the products were collected by centrifugation (5000 rpm, 15 min) and re-dispersed with an equal volume of deionized water.

### Synthesis of Au–AgNBs

The nanodot-decorated Au–AgNBs were synthesized by one-pot approach.^42^ The experiment was carried out in 100 mL conical tubes. First, 250 μL of 1% HAuCl_4_ solution was dropped into 50 mL deionized water under stirring for 1 min. After that, 850 μL of AgNO_3_ (6 mM) was introduced into the above solution and the whole solution became turbid pearl white. After introducing the 200 μL of AA (0.1 M), the mixture turned into distinct blue-violet color after about 6 s, which indicated the generation of Au–AgNBs. After ten min of magnetic stirring, the Au–AgNBs were collected by centrifugation (4500 rpm, 10 min). After the supernatant had been removed, the particles were dispersed in 20 mL deionized water for further use.

### Fabrication of Au–AgNSs immobilizing Raman reporters and labeling antibodies

10 mL of freshly synthesized Au–AgNSs were reacted with 50 μL of 4-ATP (10^−3^ mol L^−1^) or 50 μL of DTNB (10^−5^ mol L^−1^) ethanol solution under agitation for 2 h. At this time, centrifugation was performed at 7500 rpm for 20 minutes, and the precipitated Au–AgNSs were again dispersed in deionized water. After that, 20 μL PBS solution of NHS/EDC (1 : 1, 10 mg mL^−1^) was dropped into the above solution and incubated in a shaker at 37 °C for 30 min. 4-ATP/DTNB labeled Au–AgNSs with activated carboxylate terminals were achieved by centrifugation (7500 rpm, 20 min) and reacted with 5 μL of SCCA labeling antibodies (0.2 mg mL^−1^) and 5 μL of survivin labeling antibody (0.2 mg mL^−1^) at 37 °C for 2 h, respectively. 50 μL of 1% BSA was added to block bare sites to prevent nonspecific binding and shaking was continued for 1 h. Finally, Au–AgNSs@4-ATP@anti-SCCA (Au–AgNSs@DTNB@anti-survivin) were obtained after excess reagents were removed by washing process as mentioned above and then resuspended in 2 mL of 10 mM PBS solution.

### Fabrication of capture substrates

PDMS transparent substrates were made in Petri dishes, and Petri dishes were placed in ultrapure water, acetone and ethanol for ultrasonic cleaning before they were used. The mixture of 15 mL prepolymer gel and 2 mL curing agent was then placed on the surface of the Petri dish and then placed in the vacuum box for 30 min. Whereafter, PDMS was peeled off from the main body and cut into small pieces for further use. 3 mL *n*-hexane and were 4 mL Au–AgNBs colloidal solution dropped to the Petri dish, and then 2 mL ethanol was added dropwise. Au–AgNBs self-assembled at the oil–water interface to form single-layer arrays. Then, the PDMS structure was immersed in the Petri dish and the single-layer Au–AgNBs array at the oil–water interface was picked up. Then, the prepared single-layer Au–AgNBs array substrate was immersed in 2 mM DMSA solution for 4 h. PBS solution containing 20 mL NHS/EDC (1 : 1, 10 mg mL^−1^) was added for 1 h. In the process, carboxyl groups on the surface of DMSA were activated. 10 mL of SCCA coating antibody and survivin coating antibody mixed solution (200 mg mL^−1^) was conjugated on the surface of single-layer Au–AgNBs array substrate and then incubated at 37 °C for 4 h. The unconnected antibodies were rinsed out with PBS solution. Finally, the substrate was immersed in 1% BSA solution for blocking reaction and cleaned with PBS solution.

### Standard procedures of the immunoassay protocol

The sandwich immunoassay of the SCCA and survivin based on the above SERS tags and substrate was fabricated, as illustrated in Fig. 1. In the beginning, specimen solutions of SCCA and survivin with different concentrations were prepared and added to the as-prepared SERS active substrates. After that, the substrates were stored at 37 °C for 2 h to boost the interaction between antigen and antibody. Subsequently, the substrates were dipped in as-prepared SERS tags to form a sandwich immune structure and stored at 4 °C for 2 h. In each step, the substrates were rinsed three times by deionized water to remove the extra material. Ultimately, the entire sandwich structure was obtained after evaporation and SERS measurements were carried out using a Raman spectrometer in the range of 400–1800 cm^−1^. Each specimen was scanned by spectrometer (excitation wavelength: 785 nm, laser power: 5 mW, exposure time: 10 s, objective lens: 50×) at ten random positions to obtain the average SERS spectrum.

### Instrumentation

Ultraviolet-visible-near infrared (UV-vis-NIR) absorption spectra were measured using a UV-vis spectrometer (UV-3000PC, Mapada, China). Transmission electron microscopy (TEM) images were recorded by a transmission electron microscope (Tecnai 12, Philips, Netherlands). Scanning electron microscopy (SEM) images were investigated with an S-4800II field-emission scanning electron microscopy (Gemini SEM 300, Carl Zeiss, Germany). A field emission transmission electron microscope (Tecnai G2F30 S-TWIN, FEI, USA) was utilized to obtain high resolution TEM (HRTEM) images, selected-area electron diffraction (SAED) and elemental mapping images. Fourier transform infrared spectroscopy (FT-IR) analyses were carried out on a Cary 610/670 Microscopic infrared spectrometer (Varian, USA). An atomic force microscope (MFP-3D-Origin, Oxford, UK) was used to perform atomic force microscope (AFM) measurements. SERS mapping was recorded by a Raman spectrometer (Renishaw inVia, UK) with mapping step size of 1 μm and pinhole of 25 μm. All the experiments were carried out at room temperature.

### Simulation of electromagnetic field distribution

To confirm the spatial electric field distribution of as-prepared single-layer Au–AgNBs array under the irradiation of a beam of linearly polarized light, the finite-difference-time-domain (FDTD) simulation (8.11.337 version, Lumerical Solutions, Inc.) was performed as an effective approach. For the single-layer Au–AgNBs array, the simulation region was set as a unit of 4000 nm × 4000 nm × 1200 nm in 3Ds. Total-field scattered-field (TFSF) linearly polarized light waves, which were polarized in line with *X*-axis with a wavelength range of 400–900 nm, were injected into the unit cell along the −*Z* direction. Frequency-domain field profile monitors localized at *Z* = −47 nm, in the *x*–*y* plane, and *X* = 0 and 251 nm in the *y*–*z* plane, respectively, were utilized to collect field profile data of the surface and interior of single-layer Au–AgNBs array while keeping the excitation wavelength 785 nm.

## Results and discussion

### Characterization of Au–AgNSs

**Fig. 2:**
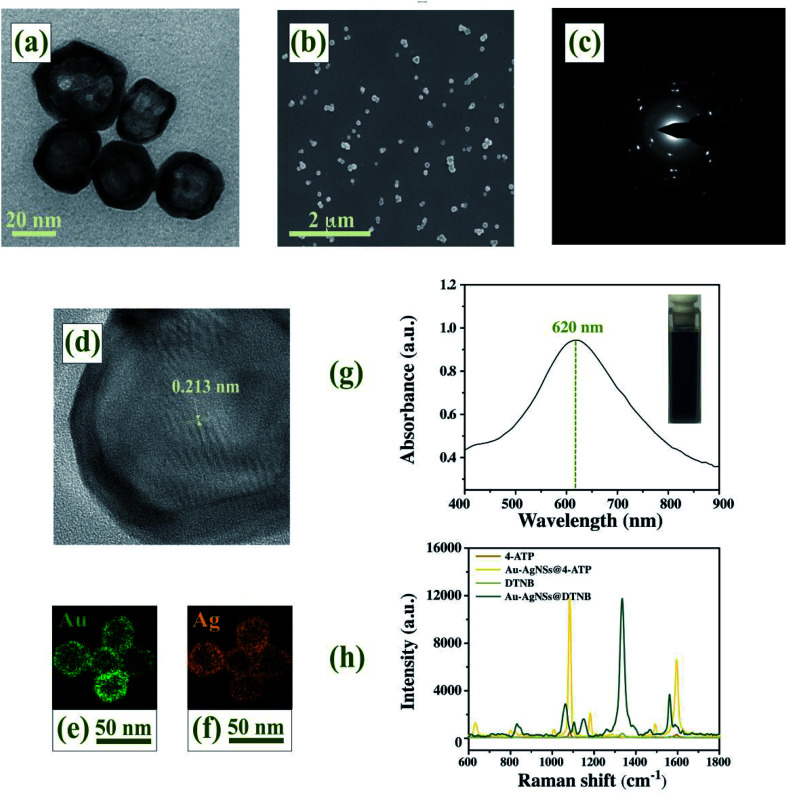
(a) TEM images, (b) SEM images, (c) diffraction pattern and (d) HRTEM images of Au–AgNSs. Au (e) and Ag (f) elemental mapping images. (g) UV-Vis-NIR absorption spectrum of Au–AgNSs. (h) SERS spectra of 4-ATP, DTNB and 4-ATP/DTNB labeled Au–AgNSs.

Representative microscopic images of Au–AgNSs were presented in TEM images (Fig. 2(a)) and SEM images (Fig. 2(b)). The nearly uniform Au–AgNSs had an average diameter of 22 nm and a wall thickness of 3.5 nm, which showed a spherical shape with smooth surfaces and thick compact walls. From the distinct lattice structures shown in the diffraction pattern (Fig. 2(c)) and HRTEM images (Fig. 2(d)), single Au–AgNSs contain twinned or polycrystalline structures with a tip lattice spacing of 0.213 nm. The Dynamic light scattering (DLS) analysis of Au–AgNSs is shown in Fig. S1 (ESI†), the mean of Au–AgNSs size was found to be 25.2 nm (size distribution range 10.25–39.2 nm). Polydispersity index measured by DLS is 0.07. Fig. S2 (ESI†) exhibited particle size distributions of Au–AgNSs from SEM image. Image-J software was used for measuring particle size. The size distribution of Au–AgNSs exhibited highest frequency at 22 ± 1.5 nm. The size measured by SEM was comparable to those obtained from DLS. The elemental mapping was conducted to acquire the elemental information on Au (Fig. 2(e)) and Ag (Fig. 2(f)), which demonstrated that the Au and Ag compositions were almost completely overlapped. From the characterization results, Au–AgNSs was a homogeneous alloy. As illustrated in Fig. 2(g), the localized surface plasmon resonance (LSPR) band of the as-synthesized Au–AgNSs appeared at 620 nm.^42^ Photographs of Au–AgNSs aqueous solution corresponding to the curves was shown in the inset. Fig. 2(h) showed that 4-ATP labeled Au–AgNSs produced prominent characteristic peaks at 1081 cm^−1^ and 1586 cm^−1^, while DTNB labeled Au–AgNSs gave rise to the SERS signals of the DTNB molecules with the representative bands at 1074 cm^−1^, 1327 cm^−1^ and 1554 cm^−1^. As a characteristic of 4-ATP, the peak at 1081 cm^−1^ exhibited the highest intensity and was used to compare the enhancement of Au–AgNSs. Similarly, the characteristic peak of DTNB at 1327 cm^−1^ was also selected. SERS enhancement factor (EF) was introduced to evaluate the Raman enhancement activity based on the following formula:^43^EF = (*I*_SERS_/*C*_SERS_)/(*I*_RS_/*C*_RS_)*I*_SERS_ and *I*_RS_ were the Raman intensities for SERS and Raman measurements, respectively; *C*_SERS_ and *C*_RS_ were Raman reporters concentrations labeled with SERS tags for SERS measurements and the concentration of Raman reporters for Raman measurements, respectively. When *C*_SERS_ = 1 × 10^−5^ M and *C*_RS_ = 10^−1^ M, with the intensities at 1081 cm^−1^ obtained in Fig. 2(h), the EF of Au–AgNSs was computed as 5.388 × 10^5^. With the intensities at 1327 cm^−1^, a value of the EF = 5.521 × 10^5^ was obtained.

### Characterization of Au–AgNBs

**Fig. 3:**
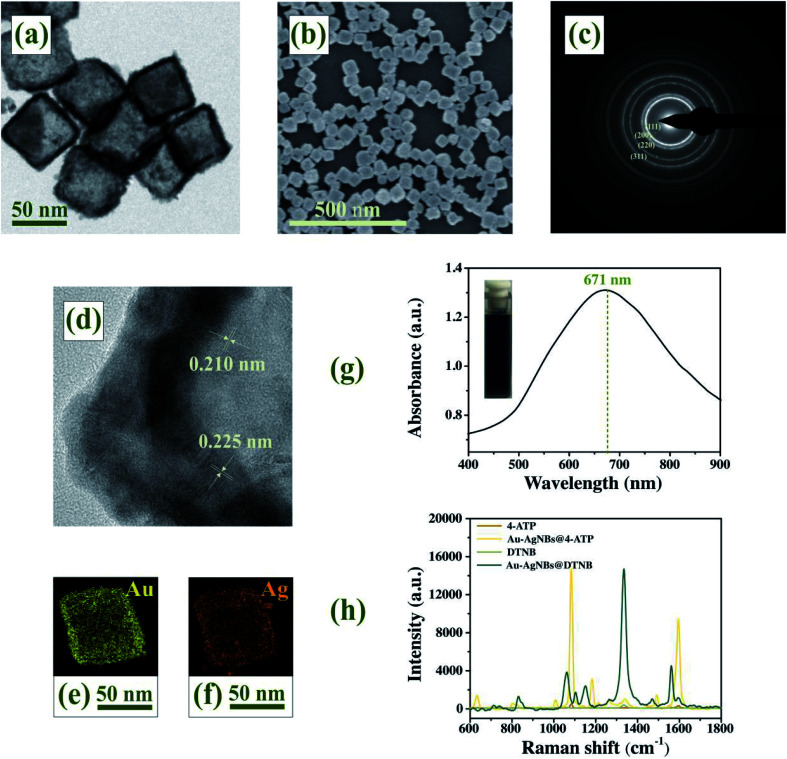
(a) TEM images, (b) SEM images, (c) diffraction pattern and (d) HRTEM images of Au–AgNBs. Au (e) and Ag (f) elemental mapping images. (g) UV-Vis-NIR absorption spectrum of Au–AgNBs. (h) SERS spectra of 4-ATP, DTNB and 4-ATP/DTNB labeled Au–AgNBs.

The morphology and dimensions of Au–AgNBs could be characterized by TEM and SEM. There was a clear contrast between the inside and outside of Au–AgNBs in the TEM image (Fig. 3(a)), that the average side length of the hollow structure is about 60 nm and the wall thickness is 4.5 nm. Fig. 3(b) showed SEM images of Au–AgNBs and indicated the large quantity was achieved using this approach. The crystal structure of Au–AgNBs could be observed by representative HRTEM and diffraction pattern. The diffraction rings in Fig. 3(c) represented the (111), (200), (220) and (311) facets of Au–AgNBs, respectively.^44^ The capping effect of Cl^−^ finally promoted the formation of cubic templates, which was consistent with halides selectively stabilizing (100) facets of Au–AgNBs.^45^ Through the change of image contrast and fringe in Fig. 3(d), it was easy to distinguish the shell and the porous interior. The measured lattice distances were 0.210 nm and 0.225 nm, which matched the structures of Au and Ag, respectively. The element distribution of Au–AgNBs was investigated by EDS images in Fig. 3(e) and (f). Compared with the interior, the Au and Ag signals on the wall were obviously enhanced, which indicating that Au–AgNBs had a hollow Au–Ag alloy structure. The obtained Ag enriched Au–AgNBs array was expected to improve SERS activity due to substantial Ag components in the nanostructures.^46^ The UV-vis spectrum of Au–AgNBs displayed a prominent broad band with a maximum at 671 nm (Fig. 3(g)), which indicated Au–AgNBs had been successfully prepared. The inset showed the photograph of Au–AgNBs solution with visible dark blue color. According to the Raman spectra in Fig. 3(h), the computed EF of 4-ATP and DTNB were estimated as 7.875 × 10^5^ and 7.477 × 10^5^, respectively. Fig. S3† recorded the SERS spectra of DTNB, DTNB on gold film and DTNB on single-layer Au–AgNBs array (ESI†). It could be seen that the signal of DTNB was very weak, while a strong SERS signal from DTNB-labeled Au–AgNBs array were observed. In addition, the SERS intensity of DTNB-labeled Au–AgNBs array was much stronger than that of DTNB-labeled gold film. These results indicated that Au–AgNBs had a good SERS enhancement effect.

### FDTD simulation

**Fig. 4:**
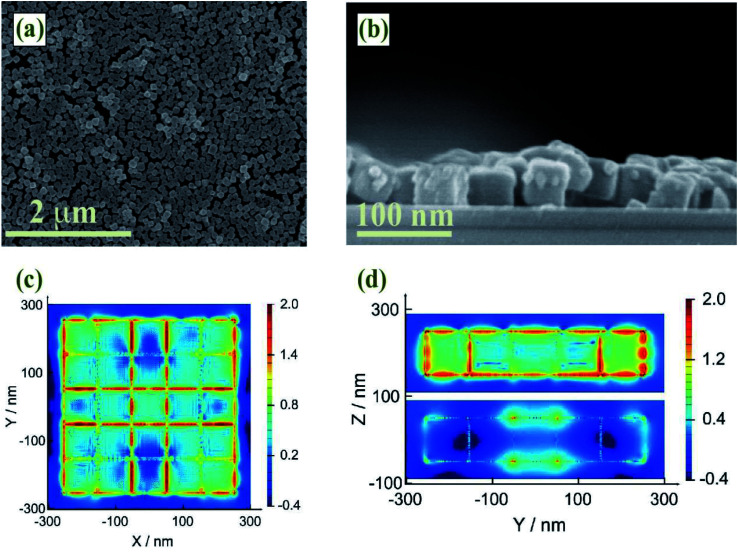
(a) SEM image of single-layer Au–AgNBs array. (b) SEM section of single-layer Au–AgNBs array. (c) FDTD simulation of single-layer Au–AgNBs array (plane). (d) FDTD simulation of single-layer Au–AgNBs array (section).

It is well known that SERS enhancement is mainly caused by the enhancement of electromagnetic fields.^47^ Since SERS enhancement could be approximated as |*E*/*E*_0_|^4^, FDTD simulations were performed To predict electric field enhancement and distribution in Au–AgNBs array substrates. All geometric parameters for simulations were consistent with the actual average size of as-prepared specimens illustrated in SEM images (Fig. 4(a) and (b)). To maintain the accuracy and effectiveness of the FDTD simulation, Au–AgNBs array was constructed with 25 Au–AgNBs in the form of a single layer (5 × 5 × 1) (Fig. 4(c) and (d)). It was easy to find that the enhanced electric field distribution was not uniform on the entire surface of single-layer Au–AgNBs array substrates, but the maximum field intensity appeared at the corners of the Au–AgNBs and the junctions of the nanodots. Depend on the results, on account of the protrusion of the Au–AgNBs array surface and the small gaps between Au–AgNBs, the laser irradiated the metal surface and produced a strong incident light electric field, which formed a considerable hot spot and amplified the Raman signal. More significantly, due to the ordered arrangement of single-layer Au–AgNBs array, strong inter-particle coupling could be easily obtained, resulting in a highly localized near-field between Au–AgNBs to enhance Raman intensity. It further confirmed that single-layer Au–AgNBs array substrates prepared by oil–water interface self-assembly method could excite out strong electric field and exhibit excellent SERS performance.

### FT-IR characterization of SERS substrate and SERS tags

**Fig. 5:**
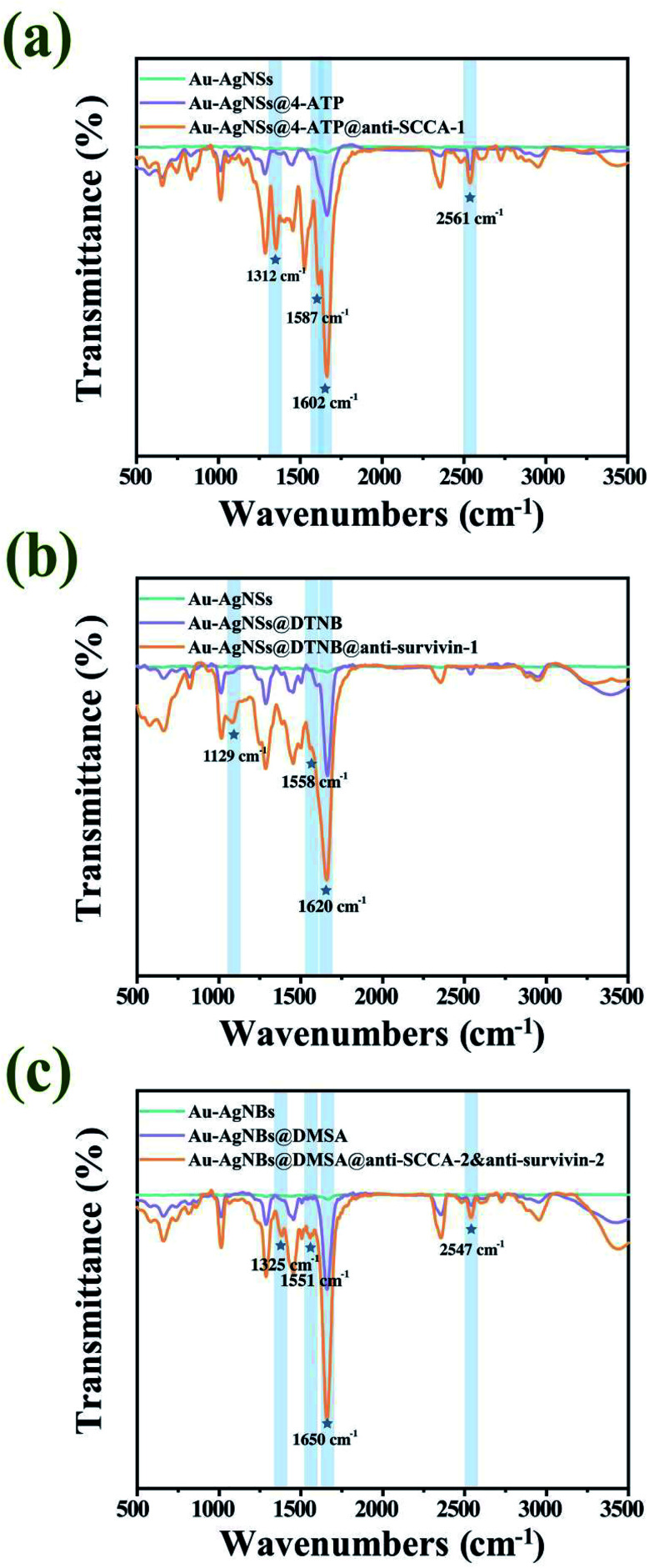
(a) FT-IR spectra of anti-SCCA-1 conjugated SERS tags. (b) FT-IR spectra of anti-survivin-1 conjugated SERS tags. (c) FT-IR spectra of anti-SCCA-2&anti-survivin-2 conjugated Au–AgNBs.

The production course for SERS substrates and SERS tags was characterized by FT-IR, as illustrated in Fig. 5. Fig. 5(a) showed the FT-IR spectra of Au–AgNSs (blue), Au–AgNSs@4-ATP (purple) and anti-SCCA-1 conjugated SERS tags (orange). There was no obvious characteristic peak on the unmodified nanomaterials. The bands at 1312 cm^−1^, 1587 cm^−1^, 1602 cm^−1^ and 2561 cm^−1^ were attributable to vibration peak of C–N, N–H amide II stretching, C=C in benzene ring and S–N stretching vibration, respectively.^48^ The existence of C–N and amide bond showed that anti-SCCA-1 was successfully conjugated to 4-ATP. Fig. 5(b) gave the FT-IR spectra of Au–AgNSs (blue), Au–AgNSs@DTNB (purple) and anti-survivin-1 conjugated SERS tags (orange). The peak at 1600 cm^−1^ appeared in spectra corresponding to the benzene skeleton vibration of C=C from DTNB,^49^ but the peaks at 1129 cm^−1^ and 1558 cm^−1^ were attributable to vibration peak of C–N and N–H amide II stretching only appeared in the spectrum of anti-survivin-1-conjugated SERS tags. The existence of amide bond demonstrated that anti-survivin-1 was successfully conjugated to DTNB. Fig. 5(c) gave the FT-IR spectra of Au–AgNBs (blue), Au–AgNBs@DMSA (purple) and anti-SCCA-2&anti-survivin-2 conjugated Au–AgNBs (orange). Among them, 1325 cm^−1^ was attributable to C–N vibration peak, 1551 cm^−1^ to N–H amide II stretching, 1650 cm^−1^ to C=O amide I stretching and the peak value at 2547 cm^−1^ corresponded to S–N stretching vibration, which indicated that the antibodies were successfully modified on the Au–AgNBs. Overall, the FT-IR spectra provided evidence that SERS tags and capturing substrates have been produced with success.

### Characterization of Au–AgNBs array substrates

**Fig. 6:**
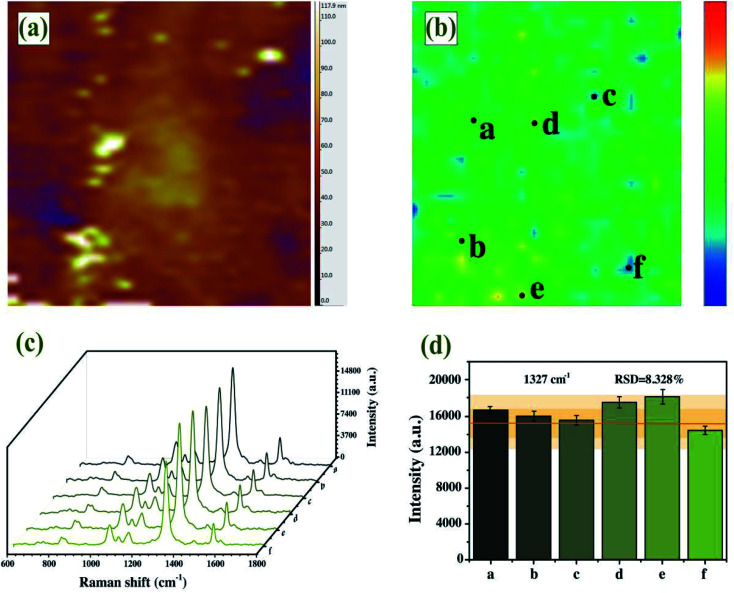
(a) AFM image of Au–AgNBs array substrate. (b) SERS mapping of Au–AgNBs array substrate measured at 1327 cm^−1^. (c) SERS spectra of Au–AgNBs array substrate and (d) peak intensities at 1327 cm^−1^ at six randomly selected spots in 40 × 40 μm^2^ area.

An ideal SERS substrate is the premise of practical SERS application and plays a significant part in uniformity, sensitivity and stability.^50^ The AFM image confirmed dense and uniform distribution of single-layer Au–AgNBs array substrate without stacking (Fig. 6(a)), with an average height of 62.3 nm. It could be seen from the AFM image that although a few regions did not contain Au–AgNBs and a few regions had a small amount of aggregation, they were uniform on a whole. SERS mapping of the substrate was measured on a randomly selected 40 × 40 μm^2^ area in the presence of DTNB molecules, as displayed in Fig. 6(b). The signals were related to the distributed amount of Au–AgNBs on the surface of the substrate. The images clearly showed a uniform distribution of SERS signal intensities over the scanned area. As shown in Fig. 6(c), six points were randomly selected on the designate area to measure the SERS spectra. The intensity histogram of the peak at 1327 cm^−1^ (Fig. 6(d)) showed the slight fluctuation of these spectra, and the relative standard deviation (RSD) is only 8.328%. It evidently demonstrated that the Au–AgNBs array substrate had an excellent uniformity.

**Fig. 7:**
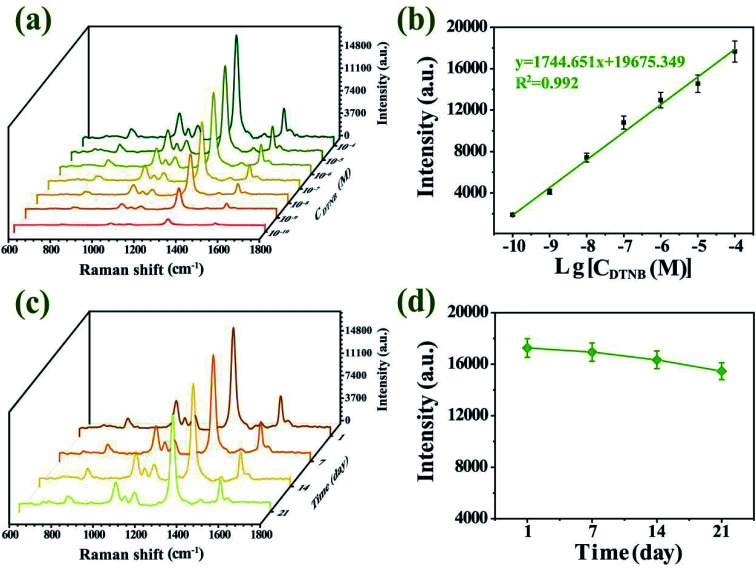
(a) SERS spectra of DTNB with different concentrations labeled Au–AgNBs array substrate. (b) The linear logarithmic relationship between the SERS signal intensity and DTNB concentration estimated by the Raman report signal at 1327 cm^−1^. (c) The average SERS spectra of DTNB labeled Au–AgNBs array substrates after being reserved for different days. (d) Linear graph corresponded to SERS intensities at 1327 cm^−1^.

In order to verify the quantitative ability and SERS sensitivity of the Au–AgNBs array substrate, DTNB was used as a target molecule. Fig. 7(a) showed the SERS spectra for diverse concentrations of DTNB in the 10^−4^ M to 10^−10^ M range, which were measured by averaging the intensities from 5 random dots. Notably, the characteristic peak at 1327 cm^−1^ was still visible even for 10^−10^ M DTNB, which indicated the promising SERS sensitivity of Au–AgNBs array substrate. The superior linear logarithmic correlation between SERS intensities and DTNB concentrations could be effectively confirmed the quantitative detection ability of the substrate (Fig. 7(b)). To evaluate the stability of developed substrate, the Au–AgNBs array substrate was reserved in a sealed container at room temperature and the SERS measurement was carried out at 1 day, 7 days, 14 days and 21 days, respectively (Fig. 7(c)). As shown in Fig. 7(d), the SERS signal changes over 10 days were within 6% of the SERS signal on day 1. On day 21, the SERS intensity maintained 92.429% of its initial SERS intensity, demonstrating the reasonable storage stability of the developed substrate.

### Characterization of selectivity and reproducibility

**Fig. 8:**
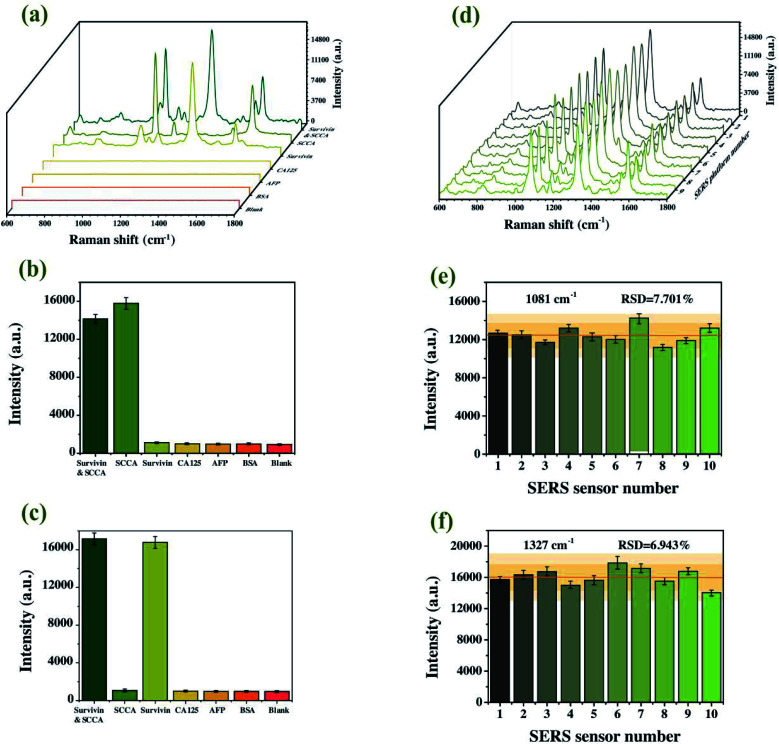
(a) The averaged spectra of the SERS signals with a range of protein interferences. Histogram of SERS intensities at 1081 cm^−1^ (b) and 1327 cm^−1^ (c). (d) The averaged spectra of the SERS signals from parallel measurements on ten independent substances. Histogram of SERS intensities at 1081 cm^−1^ (e) and 1327 cm^−1^ (f).

It is widely known that selectivity and reproducibility are two major problems for a SERS immunoassay platform. In order to evaluate the selectivity of SERS-based sandwich immunoassay platform, a control experiment was carried out with blank control and different protein specimens (SCCA, survivin, CA125, AFP and BSA) of the same concentration. As shown in Fig. 8(a)–(c), only SCCA and survivin mixtures led to a significant enhancement in Raman intensity and no unmistakable SERS signal was observed for the other specimens. Compared with the blank control group, there was no doubt that the visible SERS signals of other protein specimens were basically the background signals. As for the simultaneous detection of SCCA and survivin, two SERS signals were differentiable and showed no interference with each other. The results verified that the developed platform displayed high specificity for SCCA and survivin. Next, the reproducibility, another critical factor to investigate the property of the proposed SERS immunoassay platform, was investigated by 10 parallel tests with the SCCA and survivin specimen solution independently and Fig. 8(d) revealed that these averaged SERS spectra were almost identical. Specifically, the per-pixel mean SERS intensities at 1081 cm^−1^ and 1327 cm^−1^ of the ten individual experiments were presented in Fig. 8(e) and (f). The RSDs were calculated to be 7.701% and 6.943%, respectively, which validated the appropriate precision of the proposed method. Thereupon, the results verified the commendable practical application characteristics of the proposed SERS immunoassay platform.

### Quantitative determinations of SCCA and survivin based on SERS immunoassay platform

**Fig. 9:**
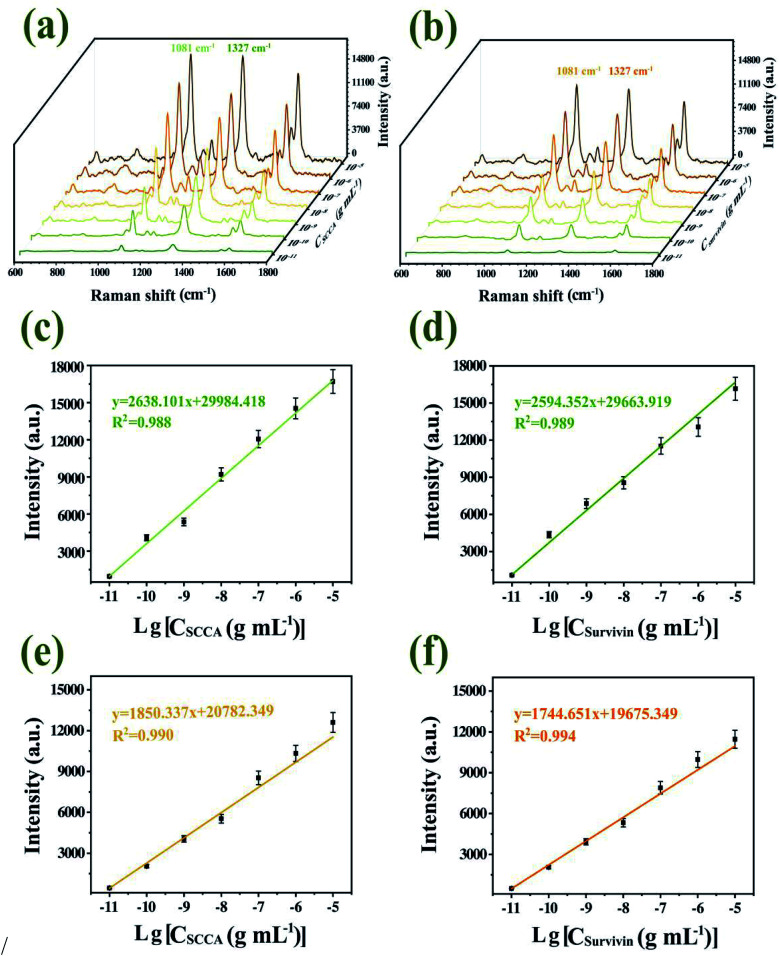
Averaged SERS spectra obtained from SCCA and survivin with different concentrations in PBS solution (a) and serum (b). Calibration curves of the corresponding peak intensities at 1081 cm^−1^ and 1327 cm^−1^ as a function of logarithm of SCCA and survivin concentration in PBS and serum, respectively (c–f).

SCCA and survivin were spiked into PBS solution and serum at different concentrations (from 10 pg mL^−1^ to 10 μg mL^−1^), followed by quantitatively detected by SERS immunoassay platform. Fig. 9(a) and (b) illustrated that with the decrease of SCCA and survivin concentration in PBS solution and serum, the Raman characteristic peak intensity of 4-ATP at 1081 cm^−1^ and DTNB at 1327 cm^−1^ decreased, which was in accordance with the fact that with a decrease in the SCCA and survivin concentration, the formation of immune complexes decreased. Further, the SERS intensities of the characteristic peaks of 1081 cm^−1^ and 1327 cm^−1^ were plotted as a function of the logarithm of SCCA and survivin concentrations, respectively, which were shown in Fig. 9(c)–(f). The figure insets displayed the linear regression equations of calibration curve and the corresponding *R*^2^ value. The limit of detection (LOD) was computed according to the regression equations by adding the signal of blank control and three standard deviations of blank control, according to the formula one shown below:^21^LOD = 10^((3×stdev−*b*)/*a*)^

The computed LODs were 5 pg mL^−1^ for SCCA and 4 pg mL^−1^ for survivin in PBS solution, while the computed LODs were 6 pg mL^−1^ for SCCA and 5 pg mL^−1^ for survivin in serum. The current detection limit for SCCA and survivin using a ELISA test were 0.17 ng mL^−1^ and 0.0625 ng mL^−1^, respectively,^51,52^ which does not always meet the requirements of clinical diagnosis. Therefore, the proposed method showed good applicability for the quantitative analysis of the two biomarkers in serum at clinically relevant concentrations.

### Practical specimens analysis

**Fig. 10:**
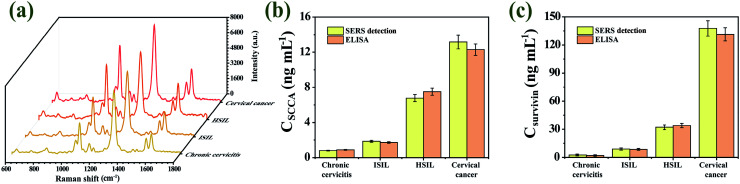
(a) The average SERS spectra of practical specimens. The concentrations of SCCA (b) and survivin (c) in practical specimens, which were detected by ELISA and the SERS detection.

Practical specimens (chronic cervicitis, LSIL, HSIL and cervical cancer patients) were monitored to validate the feasibility, reliability, and accuracy of our proposed SERS immunoassay platform for practical applications in complex biological matrixes. The SERS signals of 4-ATP and DTNB were measured for quantitatively analyzing SCCA and survivin in these practical specimens, respectively. Fig. S4(a)–(d)† showed the SERS spectra of SCCA and survivin from 40 chronic cervicitis specimens, 40 LSIL specimens, 40 HSIL specimens and 40 cervical cancer specimens (ESI†). The test results of the two methods and the RSD of each group are shown in Tables S1–S4 (ESI†). Fig. 10(a) showed the average SERS spectra of practical specimens, SERS signals from the two Raman reporters both increased with the course of the disease. The concentrations of SCCA and survivin in each practical specimen were determined by fitting these SERS intensities into the linear regression equations of calibration curve. The concentration of the two biomarkers in serum of cervical cancer patients was higher than those in HSIL, LSIL and chronic cervicitis patients. The same practical specimens were also detected by ELISA kits to assess the precision of the new method. Fig. 10(b) and (c) showed the SCCA and survivin concentrations in practical specimens measured by SERS detection and ELISA, respectively. As presented in Table 2, all RSDs were less than 20%, which indicated that the data from SERS immunoassay platform were essentially in agreement with the results obtained by ELISA. It is concluded that the proposed strategy possesses promising frontiers for early and fast diagnosis of cervical lesions.

**Table 2:** Clinical specimen determination results of SCCA and survivin

Specimen	SERS detection (ng mL^−1^)		ELISA (ng mL^−1^)		RSD [%]	
	SCCA	Survivin	SCCA	Survivin	SCCA	Survivin
Chronic cervicitis	0.803	0.298	0.897	0.271	7.820	6.711
LSIL	1.856	9.377	1.722	8.816	5.296	4.361
HSIL	6.778	32.155	7.505	34.625	7.198	5.231
Cervical cancer	13.167	137.698	12.293	131.373	4.855	3.324

## Conclusion

In summary, a high-performance SERS immunoassay platform was successfully developed for simultaneous determination of SCCA and survivin based on combining SERS tags (Au–AgNSs) with capture substrate (Au–AgNBs array substrate) prepared by oil–water interface self-assembled method. When it comes to the FDTD simulations, due to the ordered arrangement of single-layer Au–AgNBs array, the improvement of SERS activity is mainly attributed to local electric field enhancement in the gaps of Au–AgNBs. The test outcomes confirmed that the developed substrate displayed an outstanding manifestation in uniformity, sensitivity and stability and had effectively quantitative detection ability. For the quantitative analysis of the two biomarkers in serum, a linear logarithmic range from 10 pg mL^−1^ to 10 mg mL^−1^ was gained and the LODs were computed to be 6 pg mL^−1^ and 5 pg mL^−1^. Additionally, an exceptional selectivity and reproducibility of this SERS platform were demonstrated. Furthermore, this SERS immunoassay platform was applied to analyze the two biomarkers in practical specimens. The results were consistent with those of the ELISA, which made it possible to find promising applications in non-invasive cervical lesions screening in different grades.

## Ethics approval

This work was followed the ethical principles of medical research involving human body in Helsinki declaration. All serum specimens were collected with approval of the Ethics Committee of Yangzhou University School of Clinical Medicine, China.

## Conflicts of interest

There are no conflicts to declare.

## Supplementary Material

RA-011-D1RA03082E-s001
