# Supplementary material for: A sandwich SERS immunoassay platform based on a single-layer Au–Ag nanobox array substrate for simultaneous detection of SCCA and survivin in serum of patients with cervical lesions
Source: RSC Adv. 2021 Nov 16;11(58):36734–47. doi: 10.1039/d1ra03082e (PMC9043334; doi:10.1039/d1ra03082e)
Supplement: RA-011-D1RA03082E-s001 [file RA-011-D1RA03082E-s001.pdf]

Electronic Supplementary Material (ESI) for RSC Advances.
This journal is © The Royal Society of Chemistry 2021

Supporting Information

A Sandwich SERS Immunoassay Platform based on Single-Layer Au-Ag Nanoboxes Array Substrate for Simultaneous Detection of SCCA and Survivin in Serum of Patients with Cervical Lesions

Yifan Liu[1,2,3], Menglin Ran[2,3], Yue Sun[1,4,5], Yongxin Fan[1,4,5], Jinghan Wang[3], Xiaowei Cao[1,4,5*], Dan Lu[1,2,3,4*]

[1]Institute of Translational Medicine, Medical College, Yangzhou University, Yangzhou, P. R. China

[2]Department of Obstetrics and Gynecology, College of Clinical Medicine, Yangzhou University, Yangzhou, P. R. China

[3]The First Clinical College, Dalian Medical University, Dalian, P. R. China

[4]Jiangsu Key Laboratory of Integrated Traditional Chinese and Western Medicine for Prevention and Treatment of Senile Diseases, Yangzhou University, Yangzhou, P. R. China

[5]Jiangsu Key Laboratory of Experimental & Translational Noncoding RNA Research, Medical College, Yangzhou University, Yangzhou 225001, China

***E-mail:cxw19861121@163.com and ludan1968@126.com**

Results and discussion

Dynamic light scattering analysis (DLS) of Au-AgNSs

In order to investigate the particle size distribution of Au-AgNSs, we supplemented the DLS analysis. As shown in Fig. S1, the mean size was found to be 25.2 nm. The size of Au-AgNSs was mainly distributed from 10.25 nm to 39.2 nm. The size measured by DLS was comparable to those obtained from TEM (Fig. 2(a)). This result showed that the size of Au-AgNSs had good uniformity.

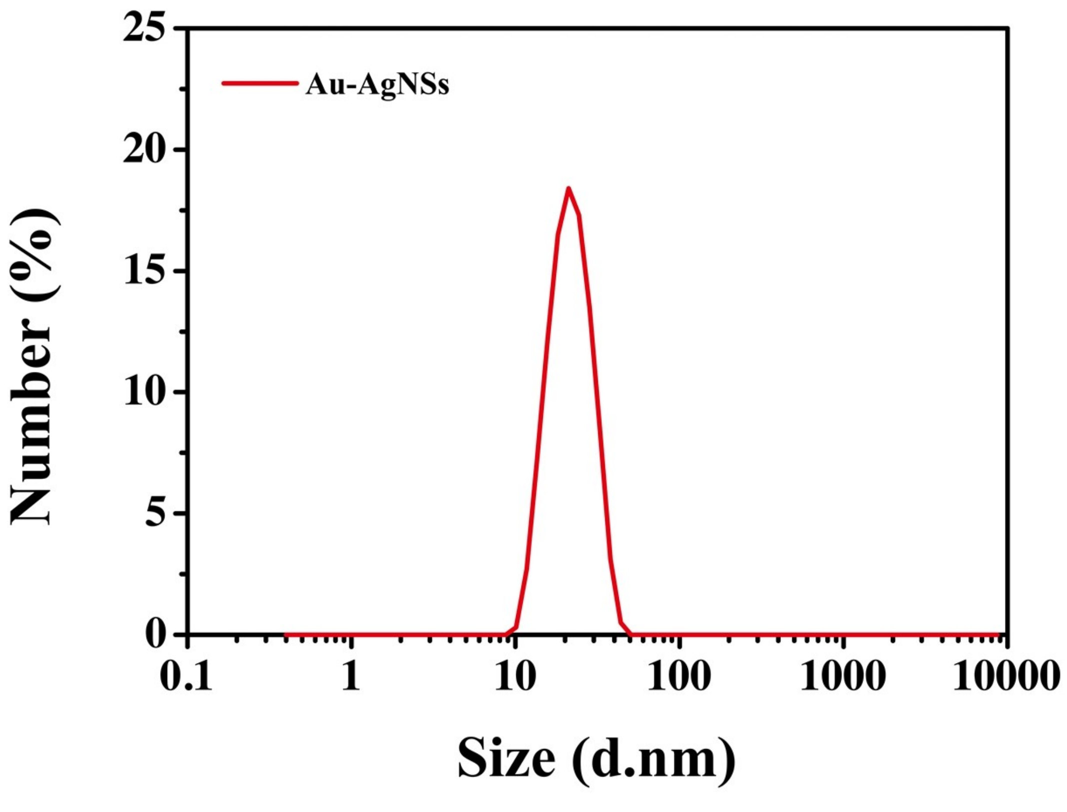

Fig. S1 Dynamic light scattering of Au-AgNSs.

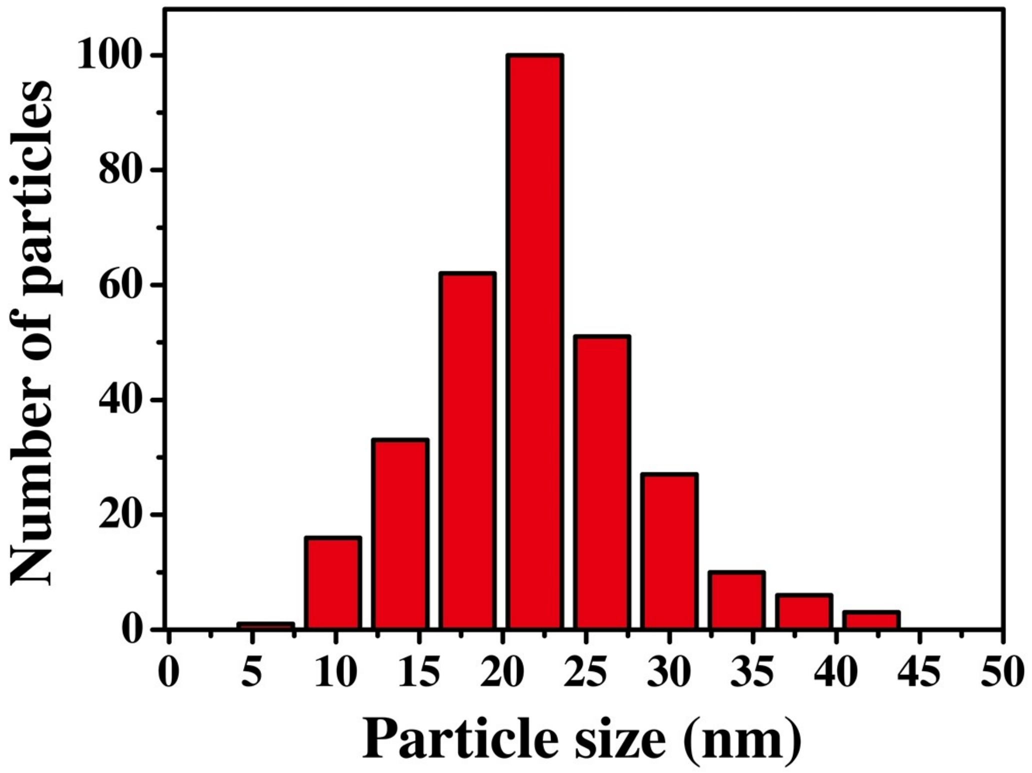

Fig. S2 Particle size distributions of Au-AgNSs from SEM.

Comparison of gold film and single-layer Au-AgNBs array

Fig. S2 recorded the SERS spectra of DTNB, DTNB on gold film and DTNB on single-layer Au-AgNBs array. DTNB was selected as Raman report molecule, which characteristic peak located at 1327 cm^{-1}. It could be seen that the signal of DTNB was very weak, while a strong SERS signal from DTNB-labeled Au-AgNBs array were observed. In addition, the SERS intensity of DTNB-labeled Au-AgNBs array was much stronger than that of DTNB-labeled gold film. The significant SERS enhancement effect may be due to the excellent surface plasmon resonance effect of

Au-AgNBs with hollow interior and porous walls, which depends on the coupling between the internal and external surface fields[1].

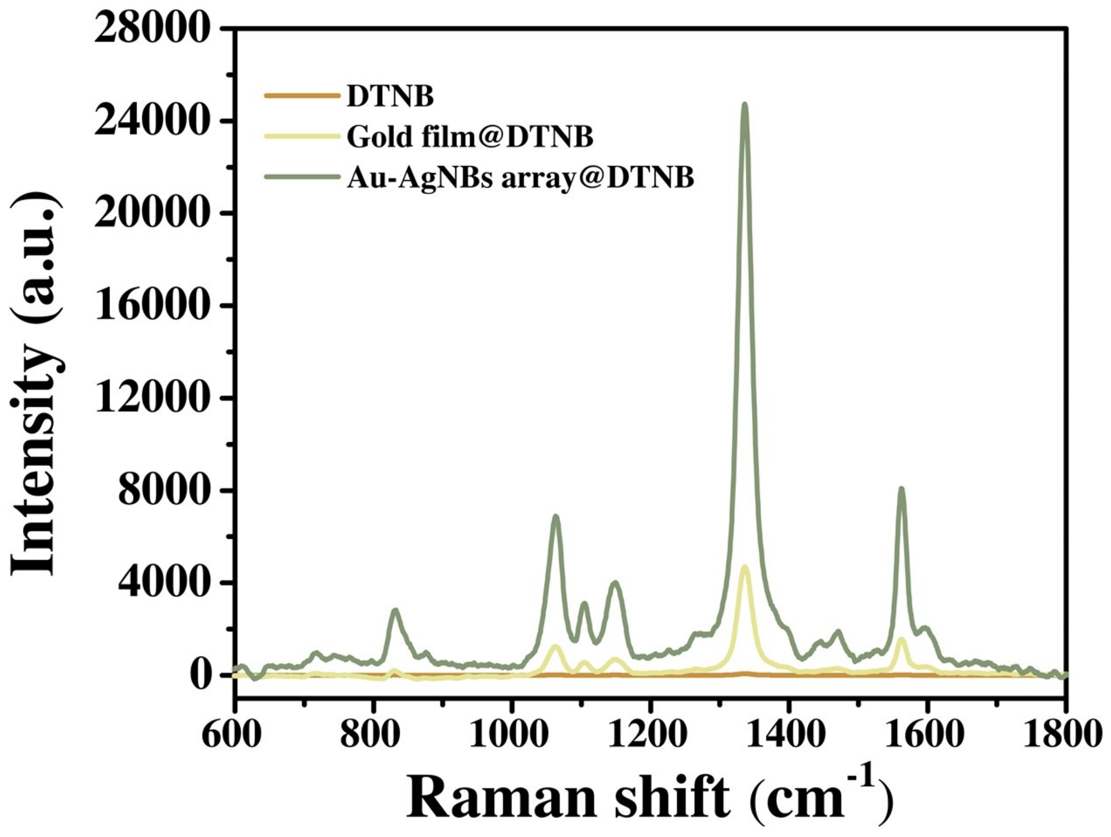

Fig. S3 SERS spectra of the DTNB, DTNB-labeled gold film and DTNB-labeled Au-AgNBs array.

Clinical application analysis

Fig. S3(a-d) showed the SERS spectra of SCCA and survivin from 40 chronic cervicitis specimens, 40 LSIL specimens, 40 HSIL specimens and 40 cervical cancer

specimens. The difference in experimental results was mainly due to the randomness of sample selection. In addition, the same samples were detected with ELISA kit to verify the accuracy of each test result. The test results of the two methods and the RSD of each group were shown in Table S1-S4. These results showed that SERS immunoassay platform had high accuracy when used to detect practical samples.

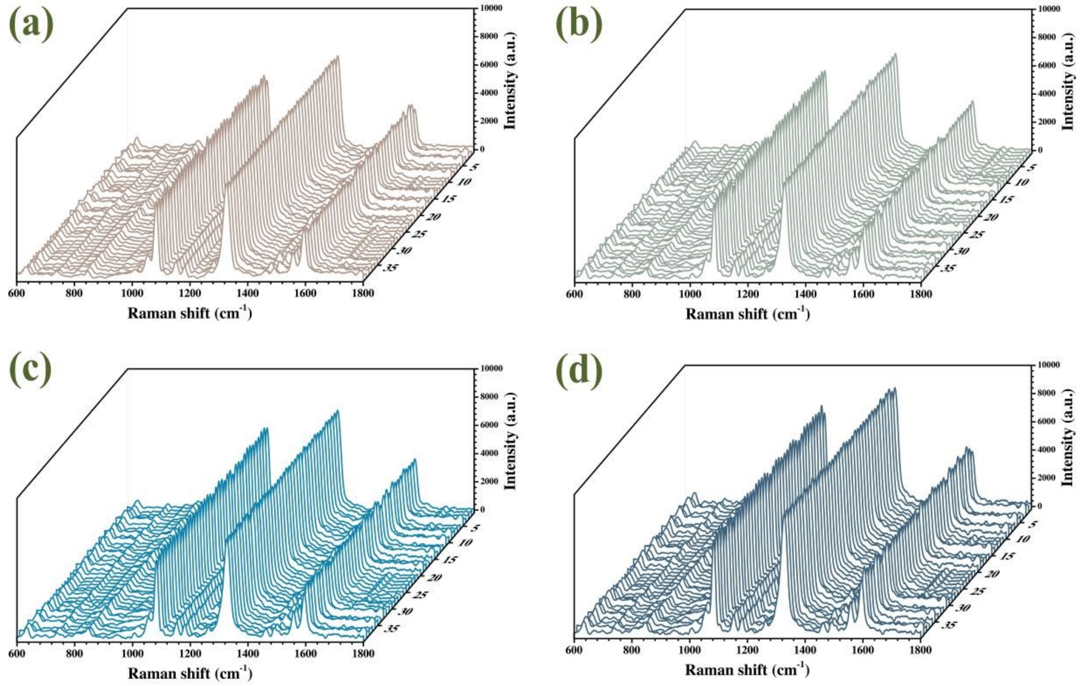

Fig. S4 (a) The SERS spectra of SCCA and survivin from **(a)** 40 chronic cervicitis specimens, **(b)** 40 LSIL specimens. **(c)** 40 HSIL specimens and **(d)** 40 cervical cancer specimens.

Table S1 Results of SERS detection and ELISA for 40 chronic cervicitis specimens

Sample	SERS detection (ng mL^{-1})		ELISA (ng mL^{-1})		RSD [%]	
	SCCA	Survivin	SCCA	Survivin	SCCA	Survivin
1	0.972	0.304	1.043	0.279	4.983	6.064
2	0.780	0.266	0.867	0.250	7.380	4.103
3	0.754	0.304	0.833	0.284	7.040	4.810
4	0.814	0.315	0.922	0.279	8.798	8.571
5	0.822	0.293	0.941	0.272	9.546	5.256
6	0.614	0.321	0.705	0.298	9.757	5.255

7	0.966	0.331	1.103	0.304	9.364	6.013
8	0.812	0.327	0.923	0.299	9.048	6.326
9	0.834	0.321	0.944	0.285	8.749	8.401
10	0.871	0.307	0.969	0.277	7.532	7.265
11	0.792	0.308	0.904	0.280	9.339	6.483
12	0.822	0.322	0.926	0.301	8.414	4.767
13	0.834	0.310	0.955	0.272	9.565	9.460
14	0.769	0.281	0.876	0.259	9.199	5.762
15	0.790	0.331	0.872	0.309	6.888	4.861
16	0.842	0.283	0.899	0.261	4.630	5.719
17	0.818	0.292	0.906	0.268	7.219	6.061
18	0.821	0.274	0.911	0.253	7.349	5.635
19	0.723	0.288	0.802	0.261	7.326	6.955
20	0.799	0.283	0.892	0.262	7.778	5.449
21	0.802	0.294	0.899	0.263	8.065	7.871
22	0.861	0.286	0.938	0.255	6.053	8.104
23	0.722	0.278	0.808	0.251	7.949	7.218
24	0.733	0.296	0.796	0.263	5.827	8.349
25	0.806	0.305	0.922	0.283	9.494	5.291
26	0.829	0.277	0.936	0.249	8.573	7.528
27	0.818	0.284	0.922	0.251	8.453	8.723
28	0.766	0.287	0.847	0.256	7.102	8.074
29	0.902	0.289	0.987	0.261	6.364	7.200
30	0.723	0.291	0.829	0.266	9.659	6.347
31	0.737	0.299	0.848	0.268	9.904	7.732
32	0.855	0.313	0.929	0.277	5.866	8.629
33	0.705	0.312	0.792	0.284	8.219	6.644
34	0.876	0.278	0.952	0.257	5.880	5.551
35	0.845	0.294	0.917	0.269	5.779	6.280
36	0.756	0.296	0.855	0.262	8.691	8.617
37	0.745	0.297	0.822	0.275	6.949	5.439
38	0.797	0.306	0.896	0.278	8.270	6.780
39	0.789	0.288	0.892	0.261	8.665	6.955
40	0.804	0.301	0.901	0.273	8.046	6.899

Table S2 Results of SERS detection and ELISA for 40 LSIL specimens

Sample	SERS detection (ng mL^{-1})		ELISA (ng mL^{-1})		RSD [%]	
	SCCA	Survivin	SCCA	Survivin	SCCA	Survivin
1	1.913	9.944	1.791	9.324	4.658	4.551
2	1.861	9.598	1.673	9.194	7.523	3.040

3	1.822	10.798	1.677	9.441	5.861	9.482
4	1.835	11.162	1.702	9.932	5.318	8.246
5	1.804	9.788	1.697	9.034	4.322	5.665
6	1.858	8.632	1.803	8.041	2.125	5.013
7	1.869	8.792	1.791	8.022	3.014	6.476
8	1.834	8.869	1.755	8.324	3.113	4.483
9	1.788	9.041	1.677	8.549	4.530	3.956
10	1.791	9.489	1.658	9.051	5.453	3.341
11	1.893	9.270	1.704	8.814	7.431	3.566
12	1.859	9.618	1.734	9.211	4.920	3.057
13	1.848	9.123	1.789	8.591	2.294	4.247
14	1.911	8.623	1.822	8.144	3.372	4.040
15	1.897	7.991	1.733	7.711	6.389	2.522
16	1.859	9.044	1.706	8.745	6.069	2.377
17	1.838	9.832	1.688	9.433	6.016	2.929
18	1.829	9.041	1.693	8.631	5.461	3.281
19	1.877	9.578	1.711	9.059	6.543	3.938
20	1.871	9.612	1.788	9.315	3.208	2.219
21	1.852	9.329	1.743	8.891	4.288	3.400
22	1.803	8.842	1.722	8.335	3.250	4.174
23	1.796	9.043	1.703	8.558	3.759	3.897
24	1.844	9.377	1.698	8.971	5.829	3.129
25	1.821	8.791	1.674	8.318	5.948	3.910
26	1.893	10.034	1.708	9.341	7.265	5.058
27	1.859	9.706	1.699	9.151	6.360	4.162
28	1.872	9.673	1.678	9.093	7.728	4.371
29	1.857	9.437	1.689	8.936	6.700	3.856
30	1.847	9.491	1.705	8.855	5.654	4.903
31	1.884	8.621	1.719	8.359	6.476	2.182
32	1.872	9.968	1.684	9.379	7.477	4.305
33	1.879	9.692	1.733	9.010	5.716	5.149
34	1.896	9.291	1.745	8.772	5.865	4.063
35	1.858	9.031	1.729	8.344	5.086	5.592
36	1.902	9.611	1.787	8.851	4.409	5.822
37	1.893	9.423	1.716	8.715	6.936	5.520
38	1.878	9.555	1.723	8.915	6.087	4.900
39	1.842	9.412	1.731	8.604	4.393	6.343
40	1.823	8.913	1.718	8.345	4.194	4.654

Table S3 Results of SERS detection and ELISA for 40 HSIL specimens

Sample	SERS detection (ng mL^{-1})		ELISA (ng mL^{-1})		RSD [%]	
	SCCA	Survivin	SCCA	Survivin	SCCA	Survivin
1	8.210	33.421	8.994	35.806	6.445	4.872
2	4.101	33.131	4.686	36.569	9.415	6.976
3	4.763	32.104	5.318	34.734	7.786	5.565
4	7.901	30.055	8.704	32.573	6.839	5.686
5	7.158	32.325	7.778	36.006	5.870	7.618
6	8.931	31.563	9.990	34.113	7.915	5.491
7	7.046	34.638	7.940	36.12	8.437	2.962
8	8.246	33.203	9.307	34.853	8.548	3.429
9	5.975	31.738	6.705	33.385	8.142	3.577
10	7.284	31.950	7.887	34.627	5.621	5.686
11	6.147	32.265	6.993	35.839	9.105	7.422
12	6.594	29.919	7.240	33.455	6.604	7.891
13	6.835	31.112	7.454	33.934	6.126	6.136
14	8.884	31.352	9.804	34.109	6.962	5.956
15	6.581	29.942	7.264	32.797	6.977	6.436
16	6.461	33.011	7.011	36.323	5.774	6.756
17	5.795	31.770	6.375	34.777	6.740	6.390
18	6.234	32.642	6.931	34.166	7.487	3.226
19	8.777	32.689	9.683	35.495	6.941	5.820
20	7.771	31.734	8.457	34.941	5.978	6.802
21	7.517	31.299	8.117	34.066	5.427	5.987
22	5.659	30.610	6.317	33.259	7.770	5.866
23	7.818	31.702	8.424	34.123	5.277	5.201
24	6.681	31.588	7.498	33.363	8.149	3.865
25	6.522	35.346	7.158	37.777	6.575	4.702
26	5.517	34.538	6.225	36.168	8.527	3.260
27	4.483	30.770	5.143	32.926	9.696	4.787
28	6.566	31.736	7.265	34.51	7.147	5.922
29	6.991	32.189	7.669	34.914	6.540	5.743
30	5.742	31.164	6.572	32.665	9.532	3.326
31	5.972	32.077	6.702	33.696	8.146	3.481
32	6.411	32.145	7.351	34.149	9.660	4.275
33	6.791	33.374	7.668	35.527	8.578	4.419
34	6.884	33.175	7.692	35.77	7.839	5.323
35	6.795	33.817	7.753	36.487	9.313	5.371
36	7.004	32.121	7.854	35.817	8.090	7.694
37	7.108	30.932	7.899	32.424	7.454	3.330
38	6.622	29.697	7.531	31.157	9.083	3.393
39	6.324	33.624	7.041	35.839	7.587	4.510

40	6.899	33.736	7.795	35.744	8.623	4.087

Table S4 Results of SERS detectionand ELISA for 40 cervical cancer specimens

Sample	SERS detection (ng mL^{-1})		ELISA (ng mL^{-1})		RSD [%]	
	SCCA	Survivin	SCCA	Survivin	SCCA	Survivin
1	11.634	139.613	10.922	132.982	4.464	3.440
2	13.396	138.850	12.393	132.033	5.497	3.559
3	12.119	137.719	11.440	131.686	4.073	3.167
4	14.547	135.314	13.302	128.103	6.320	3.871
5	13.411	138.165	12.768	132.006	3.474	3.224
6	12.336	139.838	11.460	133.908	5.203	3.064
7	12.407	139.379	11.493	133.114	5.408	3.251
8	11.596	136.028	10.776	130.136	5.180	3.131
9	10.795	136.561	9.934	129.613	5.874	3.692
10	13.270	137.288	12.585	130.494	3.744	3.588
11	14.042	138.052	13.27	130.872	3.997	3.776
12	13.414	135.627	12.417	129.052	5.458	3.513
13	12.857	136.623	12.144	130.095	4.030	3.461
14	12.383	136.730	11.344	129.996	6.193	3.571
15	11.851	135.369	10.922	129.048	5.766	3.381
16	14.894	139.967	13.736	133.305	5.720	3.448
17	11.632	137.273	11.085	131.136	3.405	3.234
18	13.836	137.404	12.982	130.323	4.501	3.740
19	13.318	138.792	12.230	133.712	6.023	2.636
20	14.163	137.337	13.114	131.730	5.439	2.947
21	15.110	136.682	14.317	130.683	3.811	3.173
22	13.588	135.934	12.988	129.005	3.193	3.699
23	13.800	136.992	13.121	130.770	3.567	3.286
24	14.174	136.475	13.089	130.166	5.626	3.346
25	13.554	140.561	12.840	135.774	3.826	2.450
26	13.687	138.353	12.871	132.628	4.345	2.988
27	12.428	139.848	11.813	133.436	3.591	3.318
28	15.383	137.123	14.505	131.585	4.152	2.915
29	14.187	136.551	13.330	130.999	4.402	2.935
30	13.173	138.914	12.137	132.829	5.789	3.167
31	12.490	136.986	11.507	130.115	5.793	3.638
32	14.073	137.947	12.881	131.997	6.254	3.117
33	13.351	138.450	12.229	132.209	6.200	3.261
34	11.931	138.472	11.288	131.883	3.916	3.447
35	14.687	139.652	13.574	132.985	5.570	3.458

36	13.231	137.969	12.429	131.230	4.420	3.540
37	13.069	135.678	12.503	129.962	3.127	3.043
38	13.235	135.927	12.076	129.051	6.473	3.670
39	11.008	138.731	10.182	132.066	5.509	3.481
40	12.629	138.740	11.747	132.201	5.117	3.413

References

1. M. A. Mahmoud and M. A. El-Sayed. *J. Am. Chem. Soc.*, 2010, **132**, 12704-12710.
